# Inside the 'Hurt Locker': the combined effects of explosive ordnance disposal and chemical protective clothing on physiological tolerance time in extreme environments

**DOI:** 10.1186/2046-7648-4-S1-A79

**Published:** 2015-09-14

**Authors:** Joseph T Costello, Kelly L Stewart, Ian B Stewart

**Affiliations:** 1 Extreme Environments Laboratory, Department of Sport and Exercise Science, University of Portsmouth, Portsmouth, UK; 2 School of Exercise and Nutrition Sciences and Institute of Health and Biomedical Innovation, Kelvin Grove, Queensland University of Technology, QLD 4059, Australia

## Introduction

Explosive ordinance disposal (EOD) technicians are often required to wear specialised clothing combinations that not only protect against the risk of explosion but also potential chemical contamination. This heavy (>35 kg) and encapsulating ensemble is likely to increase physiological strain by increasing metabolic heat production and impairing heat dissipation [1,2]. This study investigated the physiological tolerance times of two different chemical protective undergarments (2.9 kg v's 4.2 kg), commonly worn with EOD personal protective clothing, in a range of simulated environmental extremes and work intensities.

## Methods

Seven males performed eighteen trials wearing two ensembles. The trials involved walking on a treadmill at 2.5, 4 and 5.5 km.h^-1 ^at each of the following environmental conditions, 21 °C, 30 °C and 37 °C wet bulb globe temperature (WBGT). The trials were ceased if the participants' gastrointestinal temperature reached 39 °C, if heart rate exceeded 90 % of maximum, if walking time reached 60 minutes or due to volitional fatigue.

## Results

**Figure 1:**
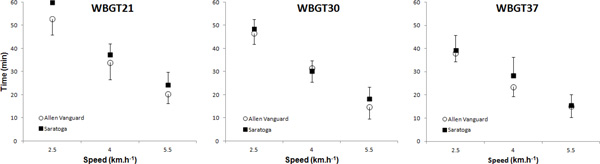
**Tolerance time (mean ± SD) in both ensembles across the different environmental conditions and work rates**.

Physiological tolerance times ranged from 8 to 60 min and the duration (Figure 1, mean difference: 2.78 min, P > 0.05) were similar in both ensembles. A significant effect for environment (21>30>37°C WBGT, P < 0.05) and work intensity (2.5>4>5.5 km.h^-1^, P < 0.05) was observed in tolerance time. The majority of trials across both ensembles (101/126; 80.1%) were terminated due to participants achieving a heart rate equivalent to greater than 90% of their maximum.

## Discussion and conclusion

This is the first study to systematically compare the physiological tolerance times of two air-permeable, charcoal-impregnated chemical protective undergarments while worn in combination with EOD personal protective clothing. Physiological tolerance times wearing these two ensembles were similar and predominantly limited by cardiovascular strain.
